# Simulation studies on electrical characteristics of silicon nanowire feedback field-effect transistors with interface trap charges

**DOI:** 10.1038/s41598-021-98182-7

**Published:** 2021-09-20

**Authors:** Yejin Yang, Young-Soo Park, Jaemin Son, Kyoungah Cho, Sangsig Kim

**Affiliations:** 1 Department of Semiconductor Systems Engineering, Korea University, Seoul, Republic of Korea; 2 Department of Electrical Engineering, Korea University, 145 Anam-ro, Seongbuk-gu, Seoul, 02841 Republic of Korea

**Keywords:** Nanoscience and technology, Nanoscale devices, Electronic devices, Physics

## Abstract

In this study, we examine the electrical characteristics of silicon nanowire feedback field-effect transistors (FBFETs) with interface trap charges between the channel and gate oxide. The band diagram, I–V characteristics, memory window, and operation were analyzed using a commercial technology computer-aided design simulation. In an *n*-channel FBFET, the memory window narrows (widens) from 5.47 to 3.59 V (9.24 V), as the density of the positive (negative) trap charges increases. In contrast, in the *p*-channel FBFET, the memory window widens (narrows) from 5.38 to 7.38 V (4.18 V), as the density of the positive (negative) trap charges increases. Moreover, we investigate the difference in the output drain current based on the interface trap charges during the memory operation.

## Introduction

Feedback field-effect transistors (FBFETs) have attracted significant attention as promising next-generation transistors owing to their low subthreshold swing (*SS*), high on/off-current ratio, and low operating voltage^1–6^. Their operating mechanism is based on a positive feedback loop that modulates the height of the potential barriers in the channel region^1–6^. Most FBFET designs have complex fabrication procedures^1,2^, or additional gate electrodes^3,5,6^. Recently, single gate-all-around (GAA) FBFETs with *p*^+^-*n*^+^-*i*-*n*^+^ Si nanowire (SiNW) channels have been proposed to reduce complex device structures and additional gate electrodes^4^. These FBFETs demonstrate high performance because the GAA SiNW structure improves the gate controllability of the potential barrier height in the channel region.

Despite the advantages of the GAA SiNW structure, the downscaling of transistors raises significant problems during the device fabrication process. The influence of trap charges located within the interface has been reported by other research groups^7–14^. The interface trap charges (ITCs) induce device degradation with respect to the *SS*, on/off current ratio, and a shift in the threshold voltage (*V*_TH_). Nevertheless, the variation in the electrical characteristics of GAA SiNW FBFETs caused by the presence of ITCs has not been reported. Hence, in this study, we analyze the variation in the electrical characteristics owing to the existence of ITCs between the silicon channel and gate oxide for planar 2-D single-gated FBFETs indicating cross-sectional view of a GAA SiNW structure via technology computer-aided design (TCAD) simulation. To demonstrate the effects of FBFETs with ITCs at the interface, we investigated the energy band, I–V characteristics, memory window, and operation. Moreover, during the memory operation, transient simulations are performed to show memory operation and the difference in output drain current (*I*_DS_) values by the ITC density (*N*_it_).

## Results and discussion

### Operating principle of the proposed FBFETs

**Figure 1:**
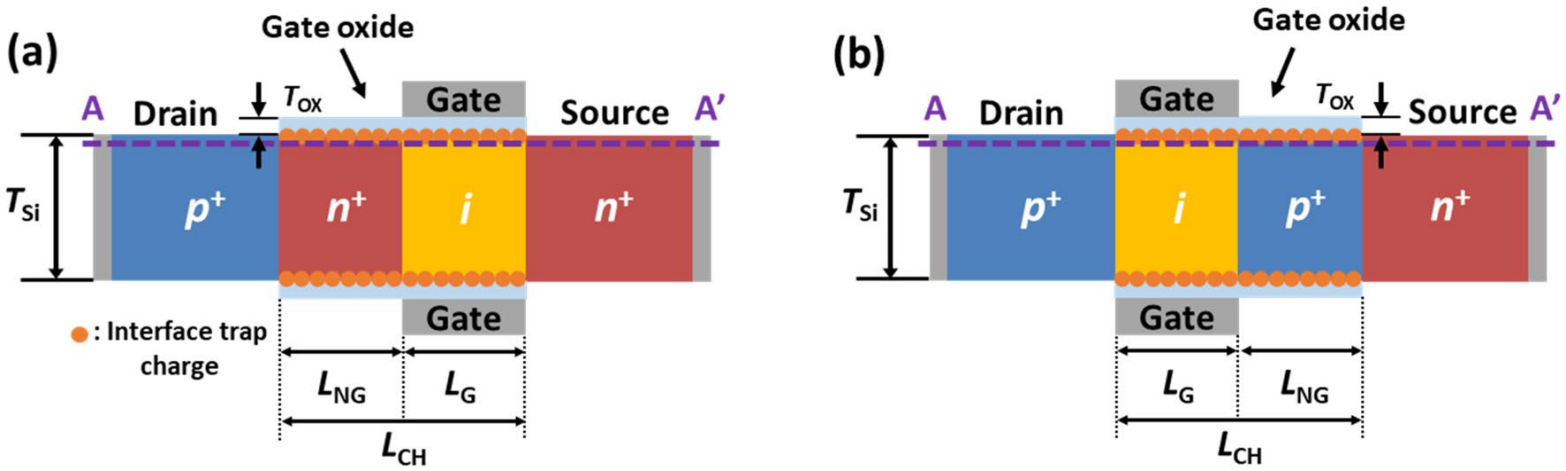
Cross-sectional view of gate-all-around (GAA) (**a**) *n*- and (**b**) *p*-FBFETs including interface trap charges between channel region and gate oxide.

**Figure 2:**
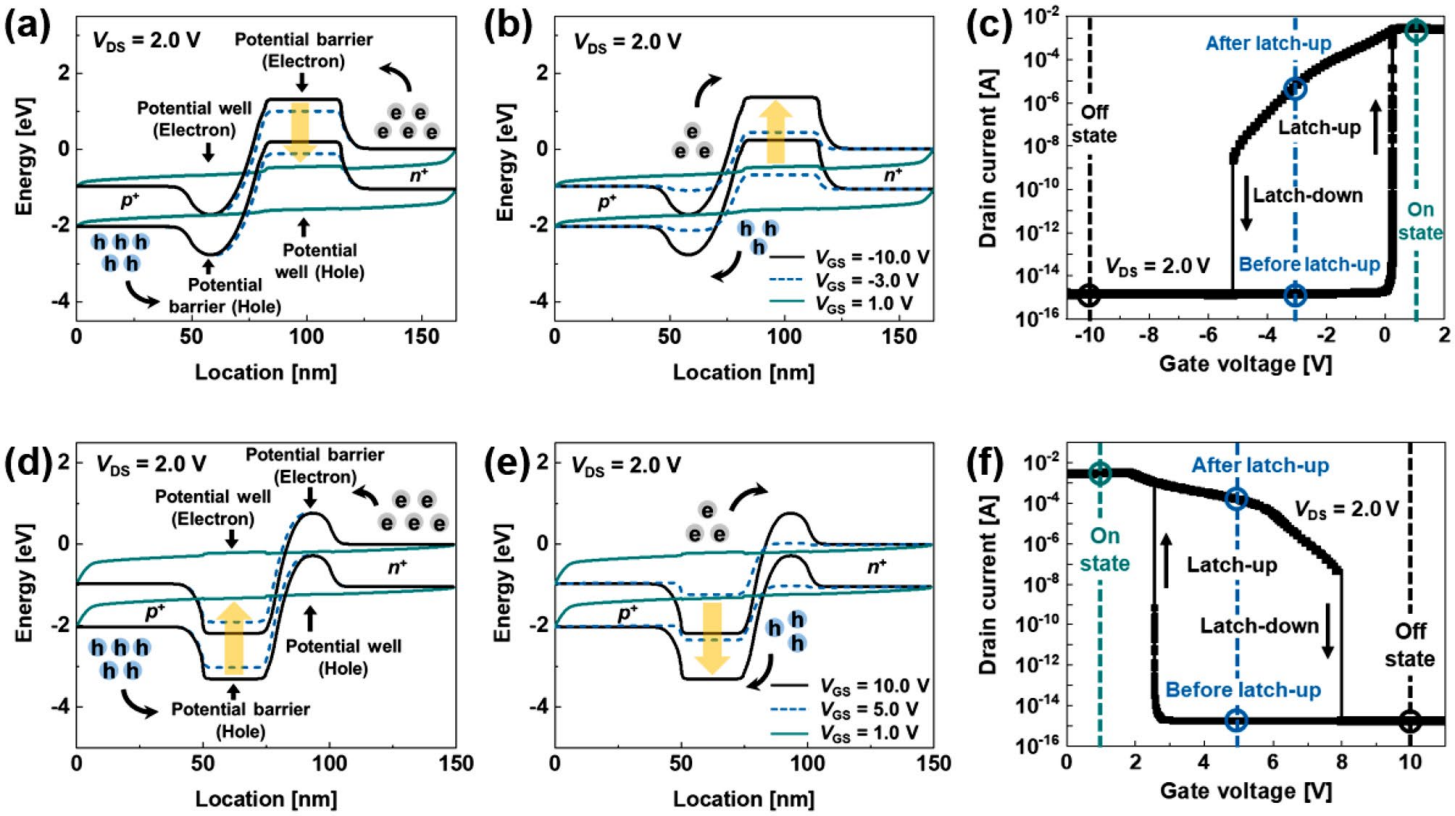
Energy band diagrams of *n*-FBFET (**a**) during positive sweeping and (**b**) negative sweeping of *V*_GS_. (**c**) *I*_DS _− *V*_GS_ transfer curve of *n*-FBFET at a *V*_DS_ of 2.0 V. Energy band diagrams of *p*-FBFET (**d**) during negative sweeping and (**e**) positive sweeping of *V*_GS_. (**f**) *I*_DS _− *V*_GS_ transfer curve of *p*-FBFET at a *V*_DS_ of 2.0 V.

The operations of the *n*- and *p*-FBFETs with single-gate electrodes shown in Fig. 1 are based on the principle of the positive feedback loop in the channel regions. Figure 2 shows the energy band diagrams of the *n*- and *p*-FBFETs and the drain current (*I*_DS_) versus the gate voltage (*V*_GS_) characteristics corresponding to the energy band diagrams without the ITCs. The *n*- and *p*-FBFETs have two potential barriers in the channel regions in the off-state under *V*_GS_ =  − 10.0 V and *V*_GS_ = 10.0 V, respectively, at a drain voltage (*V*_DS_) of 2.0 V. The potential barriers in the channel region block the injection of charge carriers. As the *V*_*G*S_ positively sweeps from − 11.0 V to 2.0 V for the *n-*FBFET and negatively sweeps from 11.0 V to 0.0 V for the *p*-FBFET at a *V*_DS_ of 2.0 V, the potential barrier heights are lowered and charge carriers are injected and accumulated in the potential wells as shown in Fig. 2a and d. Consequently, the positive feedback loop is activated by eliminating the potential barriers within a short period, and the diode current rapidly increases, which corresponds to the “latch-up” phenomenon. In the *V*_*G*S_ negative sweeping from 2.0 V to − 11.0 V for the *n*-FBFET and the *V*_*G*S_ positive sweeping from 0.0 V to 11.0 V for the *p*-FBFET, the potential barriers are regenerated by the emission of charge carriers accumulated in the channel regions as shown in Fig. 2b and e. Thus, the positive feedback loop is eliminated by regenerating the potential barriers and the diode current rapidly decreases, which corresponds to the “latch-down” phenomenon. Figure 2c and f show the *I*_DS_ − *V*_GS_ transfer curve depicting the latch-up/down phenomena and on/off state under different three gate voltages. These voltage differences between the latch-up voltage (*V*_Latch-up_) and latch-down voltage (*V*_Latch-down_) are defined as the memory window for memory operations^15^. In the following sections, we analyze how the presence of ITCs affects the positive feedback mechanisms on the *n*- and *p*-FBFETs.

### Effect of the ITCs

**Figure 3:**
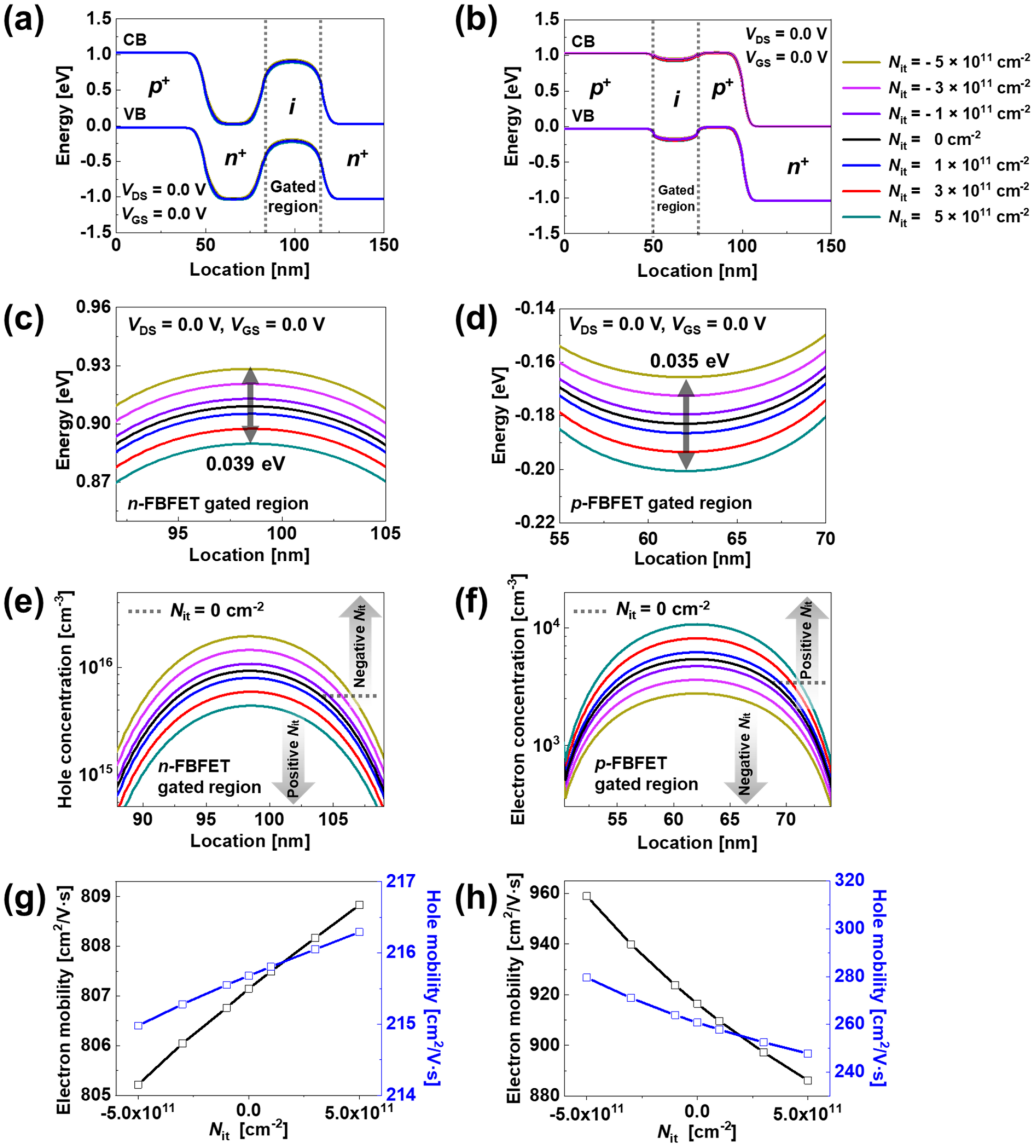
Energy band diagrams of (**a**) *n*- and (**b**) *p*-FBFETs at *V*_GS_ = *V*_DS_ = 0.0 V. Enlarged view of potential energy of (**c**) *n*- and (**d**) *p*-FBFETs in gated region. (**e**) Hole concentration for *n*-FBFET and (**f**) electron concentration for *p*-FBFET of gated channel region, respectively. Electron and hole mobility as a function of *N*_it_ for (**g**) *n*- and (**h**) *p*-FBFETs.

This section shows the DC characteristics for different values of *N*_it_ located at the interface of the *n*- and *p*-FBFETs. We analyzed the variation in electrical characteristics within the channel region by varying *N*_it_ from ± 1 × 10^11^ cm^−2^ to ± 5 × 10^11^ cm^−2^. Figure 3a and b show the energy band diagrams of *n*- and *p*-FBFETs under the no-bias condition (*V*_DS_ = *V*_GS_ = 0 V). The presence of negative ITCs at the interface increases the potential energy of the channel region, whereas the presence of positive ITCs decreases the potential energy of the channel region. Figure 3c and d show the potential energy differences based on the ITCs in the gated channel region. The reason for potential energy variation of the ITCs is the Coulomb interaction, in which ITCs attract opposite polarity charges^16,17^. Therefore, as *N*_it_ increases from 0 to − 5 × 10^11^ cm^−2^, the potential barrier height of the *n*-FBFET increases, whereas the potential barrier height of the *p*-FBFET decreases. As *N*_it_ increases from 0 to + 5 × 10^11^ cm^−2^, the potential barrier height of the *n*-FBFET decreases, whereas the potential barrier height of the *p*-FBFET increases.

To confirm the Coulomb interaction in which opposite polarity charges are induced by the ITCs at the interface, Fig. 3e and f show the hole and electron concentrations of the gated channel region in the *n*- and *p*-FBFETs, respectively; when increasing from *N*_it_ = 0 cm^−2^ to *N*_it_ =  − 5 × 10^11^ cm^−2^ in the *n*-FBFET, the hole concentration increases from 9.25 × 10^15^ cm^−3^ to 1.95 × 10^16^ cm^−3^, whereas in the *p*-FBFET, the electron concentration decreases from 5.39 × 10^3^ cm^−3^ to 2.72 × 10^3^ cm^−3^. When increasing from *N*_it_ = 0 cm^−2^ to *N*_it_ =  + 5 × 10^11^ cm^−2^ in the *n*-FBFET, the hole concentration decreases from 9.25 × 10^15^ cm^−3^ to 4.30 × 10^15^ cm^−3^, whereas in the *p*-FBFET, the electron concentration increases from 5.39 × 10^3^ cm^−3^ to 10.86 × 10^3^ cm^−3^. Consequently, the ITCs present in the interface attract opposite polarity charges to the interface owing to the Coulomb interaction.

**Figure 4:**
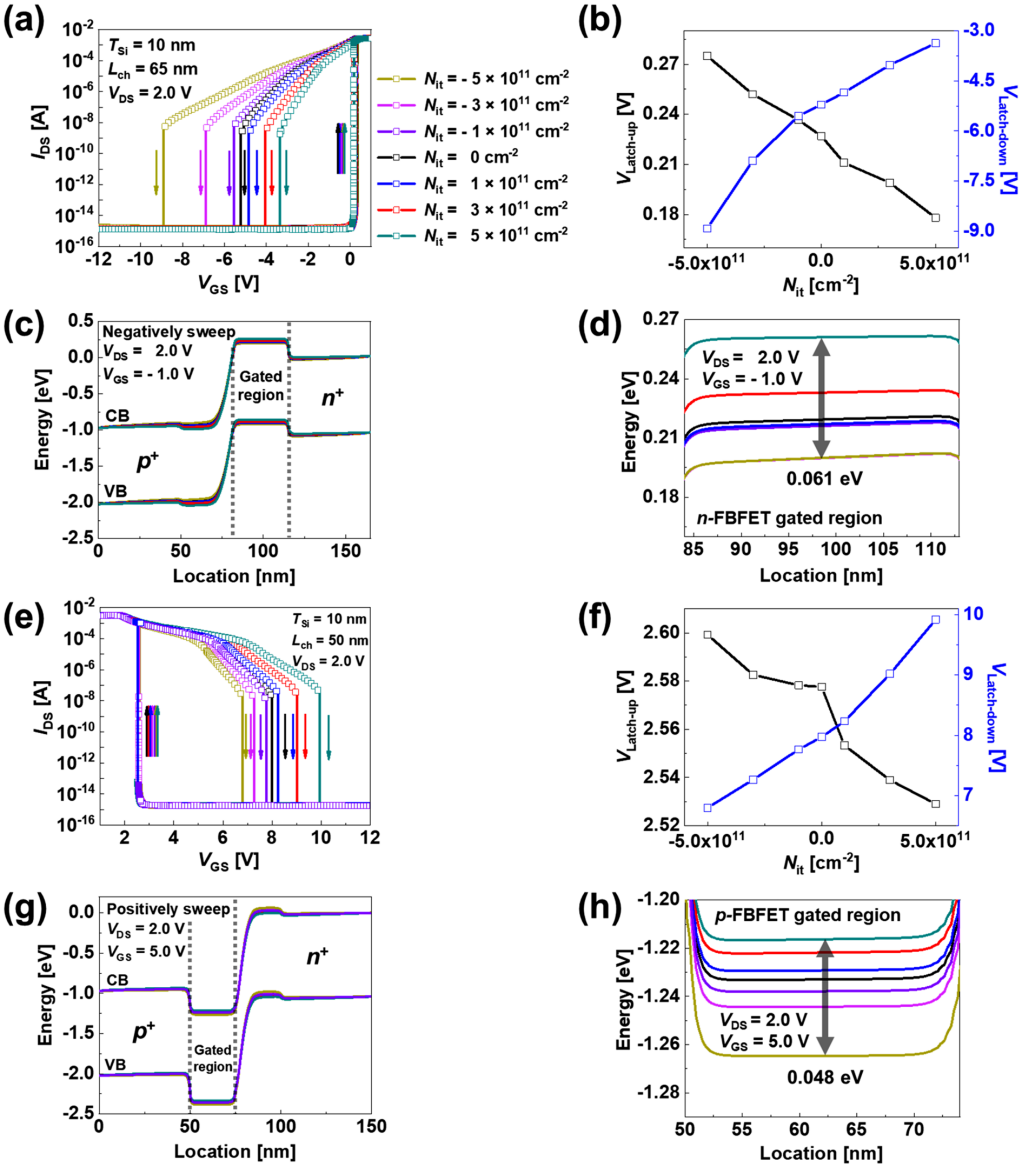
*I*_DS _− *V*_GS_ transfer curve based on different *N*_it_ of (**a**) *n*- and (**e**) *p*-FBFETs. Visual graph on *V*_Latch-up_ and *V*_Latch-down_ variation in different *N*_it_ for (**b**) *n*- and (**f**) *p*-FBFETs. Energy band diagrams of (**c**) *n*-FBFET under negative sweep and (**g**) *p*-FBFET under positive sweep. Enlarged view of potential energy of (**d**) *n*-FBFET under negative sweep and (**h**) *p*-FBFETs under positive sweep in gated region.

As mentioned above, the variations of the potential barrier height and carrier concentration based on the ITCs lead to the change in the charge carrier mobility. The higher potential barrier height prevents the injection of charge carriers in the potential well more effectively, and the carrier mobilities are degraded. On the contrary, the lower potential barrier height injects charge carriers more easily in the potential well, and the carrier mobilities are enhanced^18^. The electron mobility, hole mobility, and electric field variations under equilibrium state, off-state, and on-state condition are described in Supplementary Sect. 1. Figure 3g and h show the electron and hole mobility (*μ*_e_ and *μ*_h_) variation only based on the ITCs in the *n*- and *p*-FBFETs under equilibrium conditions (*V*_DS_ = *V*_GS_ = 0.0 V), respectively. As the *N*_it_ increases to + 5 × 10^11^ cm^−2^ with the lightly doped gated channel regions (2 × 10^15^ cm^−3^), *μ*_e_ (*μ*_h_) is enhanced to 808.83 cm^2^/V·s (216.29 cm^2^/V·s) in the *n*-FBFET, whereas *μ*_e_ (*μ*_h_) is degraded to 885.87 cm^2^/V·s (248.49 cm^2^/V·s) in the *p*-FBFET. The mobility variation by the ITCs affects the injection and accumulation of charge carriers in the channel region and shifts the *V*_Latch-up/-down_ that generates (or eliminates) the positive feedback loop. The enhanced carrier mobility owing to the higher potential barrier height generates (or eliminates) the positive feedback loop under voltage with a lower absolute value. Thus, the memory windows decrease with *V*_Latch-up_ and *V*_Latch-down_ shifts during the positive and negative sweeping of *V*_GS_. Conversely, the degraded carrier mobility owing to to the lower potential barrier height generates (or eliminates) the positive feedback loop under voltage with a higher absolute value. Therefore, memory windows increase during positive and negative sweeping of *V*_GS_. Although there is the different change between the mobility and memory window, it is notable that the carrier mobility change is consistent with the memory window change. The enhanced (or degraded) carrier mobility based on the ITCs affects the memory window width, and the memory windows are explained in next section through the DC transfer curve shown in Fig. 4a and e.

### Electrical characteristics variation by the ITCs

Figure 4 shows the DC transfer characteristics of the proposed FBFETs with positive and negative ITCs. Figure 4a shows the *I*_DS_ − *V*_GS_ transfer curves of the *n*-FBFET at a *V*_DS_ of 2.0 V. As the positive ITCs increase from *N*_it_ = 0 cm^−2^ to *N*_it_ =  + 5 × 10^11^ cm^−2^, for the *V*_GS_ positive sweeping from − 12.0 V to 1.0 V, the *V*_Latch-up_ is slightly shifted to the left. Specific *V*_Latch-up_ values corresponding to + 1 × 10^11^ cm^−2^, + 3 × 10^11^ cm^−2^, and + 5 × 10^11^ cm^−2^ are 0.21 V, 0.19 V, and 0.17 V, respectively. For the *V*_GS_ negative sweeping from 1.0 V to − 12.0 V, the *V*_Latch-down_ is shifted to the right. Accordingly, the memory window decreases from 5.47 V to 3.59 V. Accordingly, the memory window decreases from 5.47 V to 3.59 V. On the contrary, as the negative ITCs increase from *N*_it_ = 0 cm^−2^ to *N*_it_ =  − 5 × 10^11^ cm^−2^, for the *V*_GS_ positive sweeping, the *V*_Latch-up_ is slightly shifted to the right. Specific *V*_Latch-up_ values corresponding to − 1 × 10^11^ cm^−2^, − 3 × 10^11^ cm^−2^, and − 5 × 10^11^ cm^−2^ are 0.23 V, 0.25 V, and 0.27 V, respectively. For the *V*_GS_ negative sweeping, the *V*_Latch-down_ is shifted to the left. Accordingly, the memory window increases from 5.47 V to 9.24 V. Figure 4b shows the values of the *V*_Latch-up_ and *V*_Latch-down_ shifted by the ITCs in the *n*-FBFET. The enlarged *V*_*Latch-up*_ of the transfer curves is shown in Supplementary Sect. 2. Consequently, the memory windows of the *n*-FBFET narrow as the positive ITCs increase, whereas the memory windows widen as the negative ITCs increase.

Figure 4c shows the energy band diagram under the *V*_GS_ negative sweeping at *V*_DS_ = 2.0 V and *V*_GS_ =  − 1.0 V in the *n*-FBFET. This indicates that the potential barrier heights are varied by the ITCs when the same voltages are applied. A higher potential barrier height implies that the positive feedback loop is eliminated by the gate voltage with a lower absolute value^3,4,6^. The differences in the potential barrier height based on the ITCs in Fig. 4d demonstrate the tendencies of *V*_Latch-down_ during negative sweeping in Fig. 4a.

Figure 4e shows the *I*_DS_ − *V*_GS_ transfer curves of the *p*-FBFET at a *V*_DS_ of 2.0 V. As the positive ITCs increase from *N*_it_ = 0 cm^−2^ to *N*_it_ =  + 5 × 10^11^ cm^−2^, for the *V*_GS_ negative sweeping from 12.0 V to 1.0 V, the *V*_Latch-up_ is slightly shifted to the left. Specific *V*_Latch-up_ values corresponding to + 1 × 10^11^ cm^−2^, + 3 × 10^11^ cm^−2^, and + 5 × 10^11^ cm^−2^ are 2.55 V, 2.53 V, and 2.52 V, respectively. For the *V*_GS_ positive sweeping from 1.0 V to 12.0 V, the *V*_Latch-down_ is shifted to the right. Accordingly, the memory window increases from 5.38 V to 7.38 V. On the contrary, as the negative ITCs increase from *N*_it_ = 0 cm^−2^ to *N*_it_ =  − 5 × 10^11^ cm^−2^, for the *V*_GS_ positive sweeping, the *V*_Latch-up_ is slightly shifted to the right. Specific *V*_Latch-up_ values correponding to − 1 × 10^11^ cm^−2^, − 3 × 10^11^ cm^−2^, and − 5 × 10^11^ cm^−2^ are 2.57 V, 2.58 V, and 2.59 V, respectively. For the *V*_GS_ negative sweeping, the *V*_Latch-down_ is shifted to the left. Accordingly, the memory window decreases from 5.38 V to 4.18 V. Figure 4f shows the values of the *V*_Latch-up_ and *V*_Latch-down_ shifted by the ITCs in the *p*-FBFET. Consequently, the memory windows of the *p*-FBFET widened as the positive ITCs increased, whereas the memory windows narrowed as the negative ITCs increased.

Figure 4g shows the energy band diagram under the *V*_GS_ positive sweeping at *V*_DS_ = 2.0 V and *V*_GS_ = 5.0 V in the *p*-FBFET. The potential barrier height increased in the presence of negative ITCs, whereas the potential barrier height decreased in the presence of positive ITCs at the interface. The differences in the potential barrier height based on the ITCs in Fig. 4h demonstrate the tendencies of *V*_Latch-down_ during positive sweeping in Fig. 4e. Using the results of these memory window variations by the ITCs at the interface, we analyze the memory operation characteristics based on the ITCs^19^.

**Figure 5:**
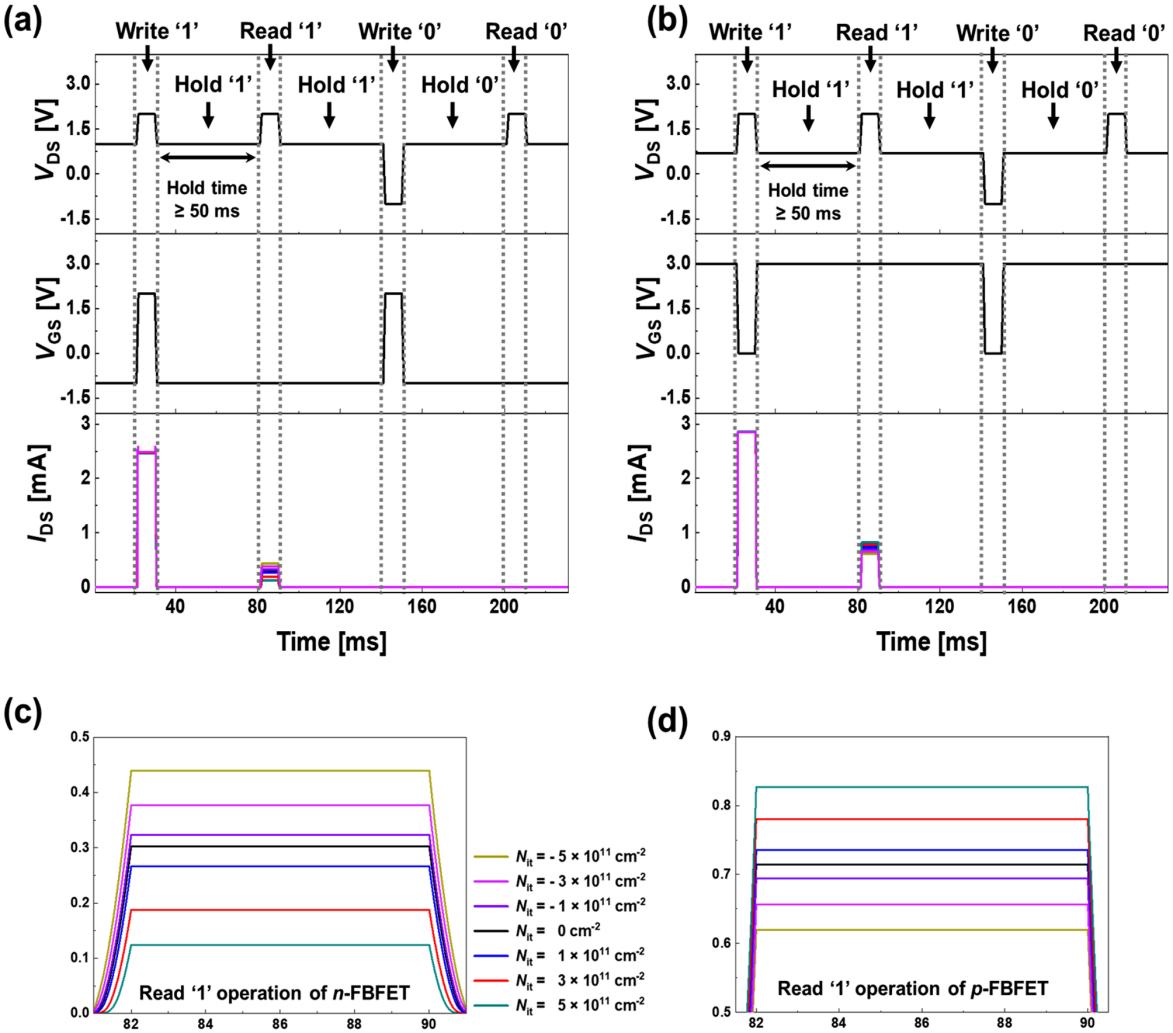
Timing diagrams for memory operation of (**a**) *n*- and (**b**) *p*-FBFETs including the ITCs. Enlarged view of output *I*_DS_ based on different *N*_it_ in (**c**) *n*- and (**d**) *p*-FBFETs during read ‘1’ operation.

Figure 5a and b show the timing diagrams for the memory operation of *n*- and *p*-FBFETs, including the positive and negative ITCs. We performed the order of write, hold, and read operations. The operation voltages are determined based on the memory window in Fig. 4a and e (for more details of the operation voltages, see Supplementary Sect. 3). Each of the *V*_DS_ and *V*_GS_ pulses has a time width of 10 ms. For the write ‘1’ operation with *V*_DS_ = 2.0 V and *V*_GS_ = 2.0 V in the *n*-FBFET and *V*_DS_ = 2.0 V and *V*_GS_ = 0.0 V in the *p*-FBFET, the positive feedback loop is generated in the channel region. The *I*_DS_ reaches approximately 2.48 mA and 2.86 mA with little difference based on the ITCs, respectively. For the hold ‘1’ operation with *V*_DS_ = 1.0 V and *V*_GS_ =  − 1.0 V in the *n*-FBFET and *V*_DS_ = 0.7 V and *V*_GS_ = 3.0 V in the *p*-FBFET, charge carriers are accumulated in the channel region while the voltages are applied. For the read ‘1’ operation with *V*_DS_ = 2.0 V and *V*_GS_ =  − 1.0 V in the *n*-FBFET and *V*_DS_ = 2.0 V and *V*_GS_ = 3.0 V in the *p*-FBFET, lowered potential barrier by injection and accumulation of charge carriers generate the positive feedback loop. The write ‘0’ operation performs recombination of accumulated charge carriers in the channel region by applying *V*_DS_ =  − 1.0 V and *V*_GS_ = 2.0 V in the *n*-FBFET and *V*_DS_ =  − 1.0 V and *V*_GS_ = 0.0 V in the *p*-FBFET. For the hold ‘0’ operation with *V*_DS_ = 1.0 V and *V*_GS_ =  − 1.0 V in the *n*-FBFET and *V*_DS_ = 0.7 V and *V*_GS_ = 3.0 V in the *p*-FBFET, there are no accumulated charge carriers in the channel region as opposed to the hold '1' operation. When the read ‘0’ operation is performed with *V*_DS_ = 2.0 V and *V*_GS_ =  − 1.0 V in the *n*-FBFET and *V*_DS_ = 2.0 V and *V*_GS_ = 3.0 V in the *p*-FBFET, the formed potential barrier makes the injection and accumulation of charge carriers difficult, which eliminates the positive feedback loop. The memory operation conditions for the *n*- and *p*-FBFETs are summarized in Table 1.

**Table 1:** Memory operating condition of *n*- and *p*-FBFETs.

Voltage	Write ‘1’	Write ‘0’	Read	Hold
***n*-FBFET**				
*V*_DS_ (V)	2.0	− 1.0	2.0	1.0
*V*_GS_ (V)	2.0	2.0	− 1.0	− 1.0
***p*-FBFET**				
*V*_DS_ (V)	2.0	− 1.0	2.0	0.7
*V*_GS_ (V)	0.0	0.0	3.0	3.0

Figure 5c and d show the *I*_DS_ values based on different *N*_it_ at the interface of the *n*- and *p*-FBFETs, respectively, during read ‘1’ operation. For the read ‘1’ operation, as positive ITCs increase to *N*_it_ =  + 5 × 10^11^ cm^−2^, the *I*_DS_ is lower in the *n*-FBFET, whereas the *I*_DS_ is higher in the *p*-FBFET. In contrast, for the same operation, as negative ITCs increase to *N*_it_ =  − 5 × 10^11^ cm^−2^, the *I*_DS_ level is higher in the *n*-FBFET, whereas the *I*_DS_ is lower in the *p*-FBFET. The reason for the difference in the current values based on *N*_it_ is that they affect the generation and elimination of the positive feedback loop. A high current value indicates that many charge carriers flow in the channel region. In addition, it indicates a low potential barrier height; thus, the memory window increases owing to the $\Delta$ *V*_Latch-up_ and $\Delta$ *V*_Latch-down_, which generate and eliminate the positive feedback loop. Conversely, a low current value indicates a high potential barrier height, hence the memory window decreases. The results correspond to the *I*_DS_ − *V*_GS_ transfer curve characteristics, as shown in Fig. 4a and e. Consequently, when negative (positive) *N*_it_ exists in the *n*-FBFET (the *p*-FBFET), the largest memory window has an advantage for their application to memory devices because the highest current flows under equally applied read voltage.

## Conclusion

We demonstrated the electrical characteristics of the proposed *n*- and *p*-FBFETs with ITCs at the interfaces. When the positive (negative) ITCs are present at the interfaces, the memory window width decreases (increases) from 5.47 V to 3.59 V (9.24 V) in the *n*-FBFET, whereas the memory window width increases (decreases) from 5.38 V to 7.38 V (4.18 V) in the *p*-FBFET. These memory window variations are depicted by potential barrier modulation owing to the presence of ITCs at the interface. Moreover, for read ‘1’ operation, the *I*_DS_ value is low (high) in the *n*-FBFET with positive (negative) ITCs, whereas the *I*_DS_ is high (low) in the *p*-FBFET with positive (negative) ITCs. Our simulation results provide information for utilization of the FBFETs with not only high performance but innate ITCs in industry.

## Methods

### Device structure and simulation models

Figure 1 shows the schematic design of the GAA SiNW *n*-channel FBFET (*n*-FBFET) and *p*-channel FBFET (*p*-FBFET) with ITCs at the Si/Al_2_O_3_ interface and the cross-sectional view proposed in this study. The channel lengths (*L*_CH_) in Fig. 1a and b were 65 nm and 50 nm, respectively. The non-gated length (*L*_NG_) and gated channel length (*L*_G_) were 1/2 L_CH_. The *p*^+^ drain and *n*^+^ source regions were 50 nm in length. The silicon channel thickness (*T*_Si_) and gate oxide thickness (*T*_OX_) were 10 nm and 2 nm, respectively. All doping concentrations of the *p*^+^-doped drain, *n*^+^-doped source, and non-gated channel region were 1 × 10^19^ cm^-3^. The gated channel region of the *n*-FBFET is lightly *p*-type doped (2 × 10^15^ cm^-3^) and that of the *p*-FBFET is lightly *n*-type doped (2 × 10^15^ cm^-3^). The work function of the gate metal is 5.0 eV.

Our simulation was performed based on the 2-D device structure of the FBFETs using a TCAD simulation (Synopsys’ Sentaurus™, Version O_2018.06)^20^. This simulation based on 2-D structure investigates and understands the overall electrical characteristics of nanoscale single-gated FBFET based on the ITCs. The models and parameters included Shockley–Read–Hall (SRH) recombination, surface SRH model for the respective interface, Auger recombination, and Slotboom bandgap narrowing. In addition, we considered the Lombardi mobility model, high-field saturation mobility model, and doping-dependence mobility. We used the default parameter of the Sentaurus Device in our simulations. In addition, to study the variation of the electrical characteristics using the effect of the ITCs, a material interface statement is used during the simulation to define the density of interface fixed charges. Moreover, we analyzed the AA’ cutline across the channel region near the interface. Different *N*_it_ values of ± 1 × 10^11^, ± 3 × 10^11^, and ± 5 × 10^11^ cm^−2^ were investigated. The *N*_it_ values refer to the published experimental data and are reported to be approximately 10^11^ cm^−2^ to 10^13^ cm^−2^^9,13^. The trap profiles followed a uniform distribution. To obtain a direct current (DC) transfer curve, we used a transient ramp. Additionally, a transient simulation was conducted to investigate the variation in memory characteristics of the ITCs.

## Supplementary Information

Supplementary Information.

## References

[1] Padilla, A. *et al*. Feedback FET: A novel transistor exhibiting steep switching behavior at low bias voltages. In *Proceedings of**2008 IEEE International Electron Devices Meeting*. 15–17 (2008).

[2] Yeung, C. W. *et al*. Programming characteristics of the steep turn-on/off feedback FET (FBFET). In *Proceedings of**2009 Symposium on VLSI Technology*. 15–17 (2009).

[3] Jeon Y (2015). Steep subthreshold swing n- and p-channel operation of bendable feedback field-effect transistors with p^(+)^-i-n^(+)^ nanowires by dual-top-gate voltage modulation. Nano Lett..

[4] Kim M (2017). Steep switching characteristics of single-gated feedback field-effect transistors. Nanotechnology.

[5] Wan J (2013). A systematic study of the sharp-switching Z2-FET device: From mechanism to modeling and compact memory applications. Solid-State Electron..

[6] Lim D (2019). Polarity control of carrier injection for nanowire feedback field-effect transistors. Nano Res..

[7] Kimukin I (2006). Surface depletion thickness of p-doped silicon nanowires grown using metal-catalysed chemical vapour deposition. Nanotechnology.

[8] Schmidt V (2006). Influence of the Si/SiO_2_ interface on the charge carrier density of Si nanowires. Appl. Phys. A.

[9] Cassé M (2010). Spectroscopic charge pumping in Si nanowire transistors with a high-κ/metal gate. Appl. Phys. Lett..

[10] Hong BH (2011). Subthreshold degradation of gate-all-around silicon nanowire field-effect transistors: Effect of interface trap charge. IEEE Electron Device Lett..

[11] Madan J (2017). Numerical simulation of N+ source pocket PIN-GAA-tunnel FET: Impact of interface trap charges and temperature. IEEE Trans. Electron Devices.

[12] Kumar N (2019). Performance assessment of the charge-plasma-based cylindrical GAA vertical nanowire TFET with impact of interface trap charges. IEEE Trans. Electron Devices.

[13] Benick, J. *et al*. Effect of a post-deposition anneal on AL_2_O_3_/SI interface properties. In *Proceedings of**IEEE Photovoltaic Specialists Conference*. 20–25 (2010).

[14] Navarro C (2019). 3-D TCAD study of the implications of channel width and interface states on FD-SOI Z2-FETs. IEEE Trans. Electron Devices.

[15] Woo S (2020). Device design of single-gated feedback field-effect transistors to achieve latch-up behaviors with high current gains. Curr. Appl. Phys..

[16] Potbhare S (2006). A quasi-two-dimensional depth-dependent mobility model suitable for device simulation for Coulombic scattering due to interface trapped charges. J. Appl. Phys..

[17] Schwank JR (2008). Radiation effects in MOS oxides. IEEE Trans. Nucl. Sci..

[18] Parihar SM (2018). Insight into carrier lifetime impact on band-modulation devices. Solid-State Electron..

[19] Bawedin M (2008). A capacitorless 1T-DRAM on SOI based on dynamic coupling and double-gate operation. IEEE Electron Device Lett..

[20] Sentaurus Device User Guide, Synopsys, Mountain View, CA, USA, (2018).

