# Supplementary material for: Simulation studies on electrical characteristics of silicon nanowire feedback field-effect transistors with interface trap charges
Source: Sci Rep. 2021 Sep 20;11:18650. doi: 10.1038/s41598-021-98182-7 (PMC8452620; doi:10.1038/s41598-021-98182-7)
Supplement: Supplementary file 1 — Supplementary Information. [file 41598_2021_98182_MOESM1_ESM.docx]

**Simulation studies on electrical characteristics of silicon nanowire feedback field-effect transistors with interface trap charges**

**Yejin Yang^1^, Young-Soo Park^2^, Jaemin Son^2^, Kyoungah Cho^2^ and Sangsig Kim^1,2*^**

^1^Department of Semiconductor Systems Engineering, Korea University, Republic of Korea

^2^Department of Electrical Engineering, Korea University, 145 Anam-ro, Seongbuk-gu,
Seoul 02841, Republic of Korea

**Supplementary Section 1.** Electron mobility, hole mobility, and electric field variations under equilibrium state, off-state, and on-state condition.

Figure S1 shows electron (*μ*_e_) and hole mobility (*μ*_h_), and electric field as a function of the location for *n*- and *p*-FBFETs. The carrier mobilities are the fastest under the equilibrium state because there is no external electric field in the gated region shown in electron and hole mobility versus location of Fig S1. The detailed description of the equilibrium state is given in the main text (see Figs. 3(g) and (h) as well). Additionally, in the equilibrium state, the electric field in performing simulation calculations are relatively low; 0.05 MV/cm for the *n*-FBFETs and 0.02 MV/cm for the *p*-FBFETs. The scattering disturbing the mobility of the charge carriers increases for the higher electric field [S1-S3]. Therefore, for the off-state, *μ*_e_ and *μ*_h_ are close to approximately zero because the high bias voltages are applied; *V*_DS_ = 2.0 V and *V*_GS_ = −10.0 V for the *n*-FBFET and *V*_DS_ = 2.0 V and *V*_GS_ = 10.0 V for the *p*-FBFET shown in Figs. S1(a) and (b), corresponding to the operating condition in Fig 2. For a high *V*_GS_ of −10.0 V (10.0 V), the scattering by a high electric field of 7.85 MV/cm (7.18 MV/cm) degrades the electron (hole) mobility in the *n*-(*p*-)FBFET. For the on-state at *V*_DS_ = 2.0 V and *V*_GS_ = 1.0 V in the *n*-FBFET and at *V*_DS_ = 2.0 V and *V*_GS_ = 1.0 V in the *p*-FBFET, *μ*_e_ (*μ*_h_) relatively is enhanced to 209.82 cm^2^/V·s (73.57 cm^2^/V·s) in the *n*-FBFET and to 294.31 cm^2^/V·s (101.88 cm^2^/V·s) in the *p*-FBFET, compared to the off-state. Specifically, in the on-state, the electric field values in performing simulation calculations are 0.47 MV/cm in the *n*-FBFET and 0.26 MV/cm in the *p*-FBFET.


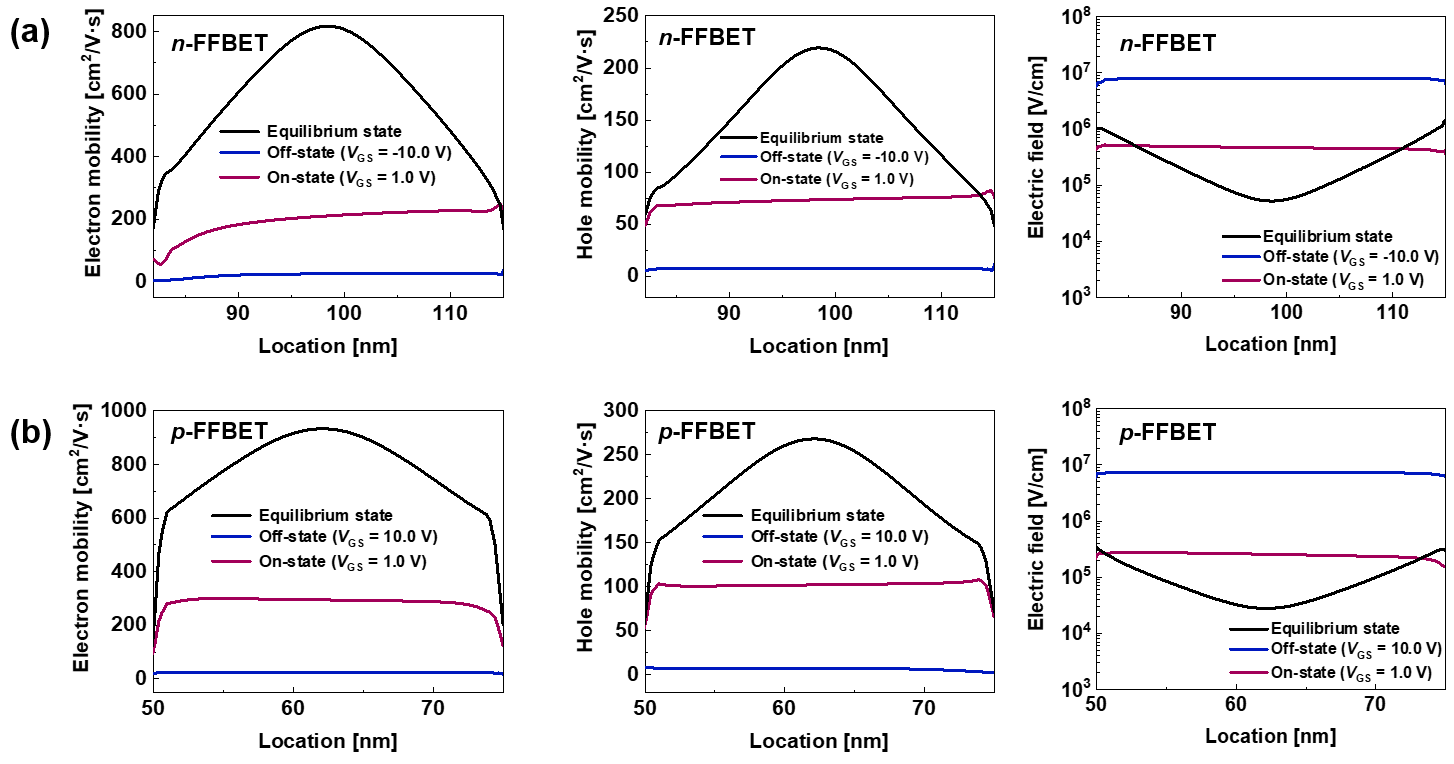


**Figure S1.** Electron and hole mobility, and electric field as a function of location for (a) *n*-FBFET and (b) *p*-FBFET without the ITCs in gated channel region.

[S1] Selberherr. S, MOS Device Modeling at 77 K. *IEEE Trans. Electron Devices*. **36**, 1464-1474 (1989).

[S2] Cheng. Y. C, *et al*. Effect of Coulomb scattering on silicon surface mobility*. Journal of Applied Physics*. **45**, 187 (1974).

[S3] Robertson. J, Interfaces and defects of high-Koxides on silicon. *Solid-State Electronics*. **49**, 283-293 (2005).

**Supplementary Section 2.** Enlarged view of the transfer curves of the *n*-/*p*-FBFETs during the *V*_GS_ sweeping.


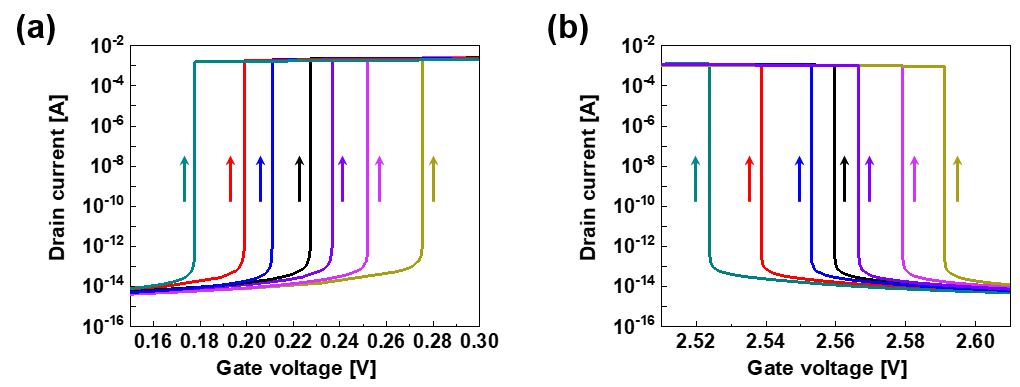


**Figure S2.** (a) Enlarged view of *V*_Latch-up_ of transfer curves for *V*_GS_ sweep range of −12.0 V and 1.0 V for *n*-FBFET. (b) Enlarged view of *V*_Latch-up_ of the transfer curves for *V*_GS_ sweep range of 1.0 V and 12.0 V for *p*-FBFET.

**Supplementary Section 3.** information on voltage conditions of “hold” and “write”.

Figure S3 shows the *I*_DS_-*V*_DS_ output and *I*_DS_-*V*_GS_ transfer curves under the write and hold conditions for the *n*-/*p*-FBFETs. The *I*_DS_-*V*_DS_ output curve shown in Fig. S3(a) indicates the write ‘0’ and ‘1’ operations for the *n*-FBFET. In the write ‘0’ operation under *V*_DS_ = −1.0 V and *V*_GS_ = 2.0 V, the bias voltages form the potential barrier, and *I*_DS_ does not flow, and in the write ‘1’ operation under *V*_DS_ = 2.0 V and *V*_GS_ = 2.0 V, the bias voltages lower the potential barrier height, and charge carriers are injected. The transfer curve indicates the hold ‘0’ and ‘1’ operations under *V*_DS_ = 1.0 V and *V*_GS_ = −1.0 V shown in Fig. S3(b). When applying hold voltages, the previous state in the potential well maintains. Likewise, the *I*_DS_-*V*_DS_ output curve shown in Fig. S3(c) indicates the write ‘0’ and ‘1’ operations for the *p*-FBFET. In the write ‘0’ operation under *V*_DS_ = −1.0 V and *V*_GS_ = 0.0 V, the bias voltages form the potential barrier, and *I*_DS_ does not flow, and in the write ‘1’ operation under *V*_DS_ = 2.0 V and *V*_GS_ = 0.0 V, the bias voltages lower the potential barrier height, and charge carriers are injected. The transfer curve shown in Fig. S3(d) indicates the hold ‘0’ and ‘1’ operations under *V*_DS_ = 0.7 V and *V*_GS_ = 3.0 V.


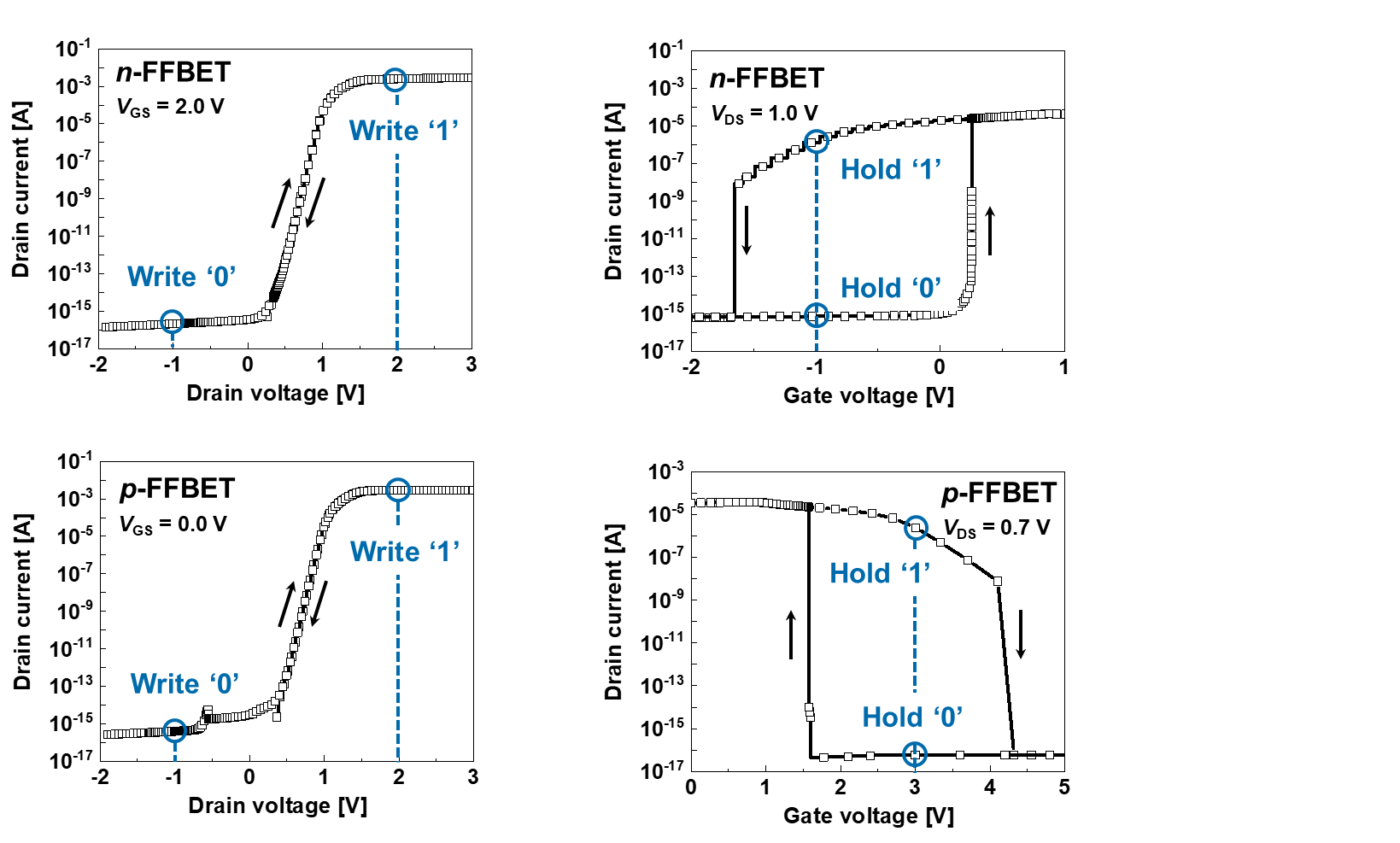


**Figure S3.** (a) *I*_DS_-*V*_DS_ output curve with *V*_GS_ = 2.0 V and (b) *I*_DS_-*V*_GS_ transfer curve with *V*_DS_ = 1.0 V for *n*-FBFET. (c) *I*_DS_-*V*_DS_ output curve with *V*_GS_ = 0.0 V and (d) *I*_DS_-*V*_GS_ transfer curve with *V*_DS_ = 0.7 V for *p*-FBFET.
